# Non‐ribosomal insights from the ribosomal P2 protein in *Plasmodium falciparum*‐infected erythrocytes

**DOI:** 10.1002/mbo3.1188

**Published:** 2021-08-22

**Authors:** Sudipta Das, Bhaskar Roy, Saswata Chakrabarty

**Affiliations:** ^1^ Asymmetric Cell Division Laboratory Division of Infectious Disease and Immunology CSIR‐Indian Institute of Chemical Biology Kolkata India

**Keywords:** cell division, channel protein complex, malaria, nuclear division, *Plasmodium falciparum*, protein oligomerization, protein trafficking, ribosomal P‐protein

## Abstract

The enormous complexity of the eukaryotic ribosome has been a real challenge in unlocking the mechanistic aspects of its amazing molecular function during mRNA translation and many non‐canonical activities of ribosomal proteins in eukaryotic cells. While exploring the uncanny nature of ribosomal P proteins in malaria parasites *Plasmodium falciparum*, the 60S stalk ribosomal P2 protein has been shown to get exported to the infected erythrocyte (IE) surface as an SDS‐resistant oligomer during the early to the mid‐trophozoite stage. Inhibiting IE surface P2 either by monoclonal antibody or through genetic knockdown resulted in nuclear division arrest of the parasite. This strange and serendipitous finding has led us to explore more about un‐canonical cell biology and the structural involvement of P2 protein in *Plasmodium* in the search for a novel biochemical role during parasite propagation in the human host.

## Graphical abstract

In malaria parasites, the 60S ribosomal stalk protein P2 translocates to the infected red blood cell surface and is potentially involved in the regulation of parasite nuclear division.

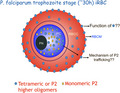

## 1 INTRODUCTION

*Plasmodium falciparum* is a unicellular eukaryotic protozoan parasite that causes malaria, a catastrophic disease in many developing countries. Malaria alone is responsible for millions of deaths annually across the globe. Drug resistance in malaria parasites is a serious issue, hence emphasizing discovering new drug targets and inventing novel small molecules that can target multiple pathways together ensuring reduced chances of resistance is one of the main challenges in the current malaria research effort. To explore new anti‐malaria drug targets, parasite ribosome can be a new focus of research interest.

The ribosome is a protein‐synthesizing nanomachine present in all living cells. In prokaryotes, ribosome comprises 30S small subunit and 50S larger subunit, whereas in eukaryotic cells 40S and 60S are the smaller and larger subunit, respectively. The building blocks of a ribosome particle are mainly rRNA and proteins. These protein molecules are exclusively associated with the ribosome and primarily engaged in their ribosomal activities but many ribosomal proteins have been discovered to perform non‐ribosomal functions at a distance in a different compartment of a cell (Jimenez‐Diaz et al., 2013; Tchorzewski et al., 2003; Volarevic et al., 2000; Wan et al., 2007; Wool, 1996). In the 60S ribosomal subunit, a structural protuberance known as stalk, which is directly involved in the interaction of the elongation factors with the ribosome during mRNA translation. The stalk is a complex of five phosphorylated proteins (P‐proteins), four small acidic proteins, and a larger protein that directly interacts with rRNA at the GTPase center during protein synthesis (Remacha et al., 1995). In eukaryotes, there are three types of P proteins, P0, P1, and P2. For example, in *Saccharomyces cerevisiae*, acidic P1⍺ and P1 β interact with P2β and P2⍺, respectively, to form [(P1⍺‐P2β)‐P0‐(P1β‐P2⍺)] pentameric stalk (e Remacha et al., 1995). In‐plants, an additional P protein, P3, has been discovered to be a part of plant ribosomal stalk (Kang et al., 2016). The stoichiometry of P1 and P2 differs in different organisms and is found to be in a constant exchange between the ribosome and cell cytoplasm (Zinker & Warner, 1976). In yeast and human cell lines, depletion of P2 leads to an instantaneous degradation of P1 (Martinez‐Azorin et al., 2008; Nusspaumer et al., 2000). In *Saccharomyces cerevisiae*, P0 null strain is lethal, whereas P1/P2 or P1‐P2 null strains do survive, but the growth rate diminishes significantly indicating their essential nature in cell survival (Remacha et al., 1992; Rodriguez‐Mateos et al., 2009; Santos & Ballesta 1995). These uncanny properties of ribosomal P‐proteins have sparked the enthusiasm to investigate whether P‐proteins in eukaryotic apicomplexan human parasites (e.g., *Plasmodium*) have any extra‐ribosomal indispensable functions which can be targeted for better therapeutic interventions. In the malaria‐endemic area, the serum of the malaria immune person has been detected with antibodies against anti‐*P*. *falciparum* P0 protein quite extensively and exclusively (Chatterjee et al., 2000; Lobo et al., 1994; Singh et al., 2002). To explore the possibility of a novel anti‐malaria intervention using P‐proteins, in a differential immunoscreen, *Plasmodium falciparum* 60S stalk ribosomal protein P0 (PfP0) was identified as a protective protein and subsequently localized on merozoite surface possibly involved during red blood cell invasion as understood using growth inhibition assay (GIA), (Lobo et al., 1994; Goswami et al., 1997). In *P*. *falciparum* ribosomes, P0 interacts with P1 and P2 to form the pentameric stalk [(P1–P2)‐P0‐(P1–P2)] (Francisco‐Velilla & Remacha, 2010; Gonzalo & Reboud, 2003; Hanson et al., 2004; Santos & Ballesta, 1994), which is required in the GTPase elongation center (Diaconu et al., 2005; Uchiumi & Kominami, 1992). While exploring eccentric properties of P0 proteins in *Plasmodium*, a puzzling phenotype of acidic ribosomal protein P2 was discovered (Das, et al., 2012a). The ribosomal P2 protein of *Plasmodium falciparum* translocates to the infected red blood cell (iRBC) surface as an SDS‐resistant oligomer and appears to play a pleiotropic role at the late trophozoite/early schizogonic stage in iRBCs (Das, et al., 2012a; Das et al., 2012b). Here, we summarize recent discoveries of *Plasmodium* P2 protein and highlight key insights and questions that need to be pondered to understand eccentric P2 biology in malaria parasites.

## 2 *PLASMODIUM* PARASITES APPEAR TO REQUIRE P2 ON THE INFECTED RED CELL SURFACE AT THE TROPHOZOITE/EARLY SCHIZOGONIC STAGE

During the late trophozoite/early schizogonic stage, SDS‐resistant P2 oligomers have been localized on the surface of iRBCs (Das et al., 2012a). Blocking the accessibility of oligomeric P2 on the iRBC surface using highly specific monoclonal antibodies resulted in nuclear division arrest of the parasite (Das et al., 2012a). In the arrested parasites, the blockage of import of fluorescently labeled lipid molecule FM4‐64 has been observed. It may be due to the degradation of lipid importing structures called tubovesicular network (TVN); Das et al., 2012a; Haldar et al., 2001; Lauer et al., 1997; Tamez et al., 2008). Washing off antibodies from the *P. falciparum* culture medium resulted in the reformation of TVN structures, the continuation of parasite nuclear division, and the completion of schizogonic processes of arrested parasites (Das et al., 2012a), indicating a potential non‐ribosomal role(s) of oligomeric P2 protein on the iRBC surface. The P2 oligomers on the iRBC surface appear to be SDS‐resistant homo/hetero tetrameric in nature (Das et al., 2012a; Das et al., 2012b) which did not resolve to a monomer in SDS containing reducing polyacrylamide gels. However, contrary to the iRBC surface, P2 protein in the parasite cytoplasm is predominantly present as a monomer indicating a possible role of SDS resistant oligomerization and oligomeric form of P2 on the iRBC surface at the trophozoite stage of parasite growth. Hence, oligomeric P2 on the iRBC surface appears to be required for the progression of the parasite growth and development. The indispensability was further confirmed as the P2 gene was found to be refractory to deletion in *Plasmodium berghei* (Bushell et al., 2017; Das et al., 2012a) and recently shown in *Plasmodium falciparum* (Zhang et al., 2018). Apart from the iRBC surface, P2 protein has also been localized on the surface of *Plasmodium falciparum* (Pf) and tachyzoite surface of *Toxoplasma gondii* (Tg) as either monomer or as an oligomer and have been implicated for host cell invasion using growth inhibition assay (GIA) (Sudarsan et al., 2015).

## 3 P2 OLIGOMERIZATION IN THE PARASITE CYTOPLASM APPEARS TO BE ONE OF THE PREREQUISITE STEPS FOR TRANSLOCATION TO THE HOST CELLS

Effector proteins in *P*. *falciparum*‐infected RBCs translocate to the host cells and render the host cell membrane rigid and promiscuous for solutes and other macromolecules (Goldberg & Cowman, 2010; Hiller et al., 2004; Russo et al., 2010). A well‐defined class of export proteins possesses a *Plasmodium* export element (PEXEL) or vacuolar targeting signal, a pentapeptide consensus sequence (RxLxE/Q/D) located 25–30 amino acids downstream of the host‐targeting signal sequence (Desai, 2012, 2014; Marti et al., 2004; Spillman et al., 2015). The cleavage of the PEXEL motif by the ER‐resident aspartic proteases, plasmepsin V is required to destine the export proteins to the host cells (Boddey et al., 2010; Osborne et al., 2010; Russo et al., 2010; Tarr et al., 2013). However, the protease cleavage of plasmepsin V does not appear to be the sole deciding factor for translocation into the host cells as PI(3)P binding to the effector proteins in the ER upstream of the PEXEL motif seems to be a prerequisite step for translocation in addition to the plasmepsin V cleavage (Bhattacharjee, et al., 2012a; Bhattacharjee, et al., 2012b; Bhattacharjee, et al., 2012c; Haldar, 2016; Hsiao et al., 2013). Another class of effector proteins, PEXEL negative exported proteins (PNEPs), do not contain PEXEL motif but still can translocate to the host cells (Bhattacharjee, et al., 2012a; Haase et al., 2009; Heiber et al., 2013; Jani et al., 2008; Pachlatko et al., 2010; Saridaki et al., 2009; Spycher et al., 2006). The first 20 amino acids at the N terminus of PNEPs without a classical “N” terminal signal sequence are sufficient to export non PEXEL effector proteins to the host cells (Haase et al., 2009; Saridaki et al., 2009) indicating a general export property of PNEPs. However, effector proteins of both the classes, PEXEL dependent and independent, do unfold in the vacuolar space before they traverse through the PTEX complex (Desai & Miller, 2014; Garten et al., 2018; Gruring et al., 2012; Ho et al., 2018).

The process of oligomerization of P2 in the parasite cytoplasm precedes the localization on the iRBC surface. Out of several oligomeric species in the parasite cytoplasm at the trophozoite stage, only homo/hetero tetrameric species of P2 appear to translocate to the iRBC surface through iRBC cytoplasm (Das et al., 2012a). Neither ghosts from the iRBC surface nor the iRBC cytoplasm showed the presence of monomeric P2 indicating that the oligomerization in the parasite cytoplasm could be initial one of the prerequisite steps in the export mechanism of P2. In the 48 h life cycle in iRBCs, the oligomerization window of P2 in the parasite cytoplasm approximately starts at around 22–24 h post‐merozoite invasion (PMI) and continues till 34–36 h PMI at late trophozoite to the early schizogonic stage (Das et al., 2012a). Before 22–24 h and after 34–36 h PMI, there were no oligomeric species of P2 observed in the western blot experiment (Das et al., 2012a; Das et al., 2012b) indicating tight regulation of oligomerization in that window of time and a possible link between oligomerization and export. After that window, oligomerization diminishes possibly due to the secretion of P2 into the culture supernatant or digestion. Immunofluorescence assay (IFA) using E2G12 after 34–36 h PMI does not seem to suggest a possibility of P2 recycling back to the parasite from the host cells as iRBC cytosol did not show any P2 staining (Das et al., 2012a).

P2 does not contain any PEXEL motif and also it is devoid of classical export signal, hence it comes under the PNEP class and possibly translocate through an unknown pathway. Transient transfection with P2‐GFP followed by immunoblotting does not seem to suggest any protease processing of P2‐GFP before export into the host cells as the molecular mass of P2‐GFP did not deviate from the theoretical mass (Das et al., 2012a). In addition to oligomerization, the probable route of P2 translocation might have PI(3)P binding and unfolding of the P2 tetramer/oligomer before PTEX traversing.

## 4 IN SOLUTION, P2 FORMS A STABLE TETRAMER WHICH IS A MOLTEN GLOBULE IN NATURE HAVING HYDROPHOBIC POCKETS ON THE SURFACE

NMR experiments in solution distinctly revealed that recombinant P2 protein has a high propensity to oligomerize and tend to form aggregates by self‐association at a millimolar concentration (Mishra et al., 2012, 2014a). Further exploration using circular dichroism (CD) and solution NMR has also revealed that the recombinant monomeric species of P2 is predominantly α helical but molten globule in nature and the “C” terminal region is intrinsically disordered in structure (Mishra et al., 2014b, 2015). At physiological pH 7.4, the thermodynamic stability of the monomer shifts toward tetramerization maintaining the molten globule nature of each monomer in the tetramer (Mishra et al., 2015), thereby keeping the flexibility of the entire tetrameric complex. Urea denaturation of recombinant P2 followed by residue level interrogation using NMR further revealed that two monomeric P2 molecules associate to form a dimer and two such dimer molecules packaged closely at their N terminus having α helices to form the tetramer (Mishra et al., 2015). 2D ^1^H – ^15^ N HSQC spectra of the native deuterated tetrameric form of P2 exhibited hydrophobic surface/pocket mostly contributed by the N terminal α helices as shown using 8‐anilinonaphthalene‐1‐sulfonic acid (ANS) (Mishra et al., 2015) indicating that P2 tetramer could provide sites for intermolecular association in an aqueous environment and may have the possibility to bind non‐polar molecules in its natively localized environment, that is, iRBC surface. Between the *P*. *falciparum* P2 and the human P2, there is 69% amino acid sequence homology but they differ in their oligomerization pattern and behavior as the human P2 at physiological pH forms a stable dimer (Grela et al., 2017). However, in the same condition, *P*. *falciparum* P2 forms a molten globule tetramer indicating some functional implications of tetrameric P2 on the surface of iRBCs at the late trophozoite stage. In a recent discovery, it has been demonstrated that P2 tetramers stabilize themselves on the iRBC surface by interacting with RBC Band3 protein where N terminal 70 amino acids of P2 interact to form the oligomers and associate with Band 3 protein (Mishra et al., 2020).

## 5 WHAT COULD BE THE POSSIBLE FUNCTION OF OLIGOMERIC/TETRAMERIC P2 ON THE iRBC SURFACE?

The selectivity of the infected red blood cell membrane is compromised due to the translocation of several effector proteins into the host cells. Virulence factors and channel proteins both are predominant components of the exportome which plays important role in disease biology and solute uptake, respectively. After the erythrocyte invasion by malaria parasites, induction of a broad specificity channel known as the new permeability pathway (NPP) into the host cells renders the red blood cell membrane non‐selective and promiscuous for a range of small‐molecule solutes including ions (Kirk, 2015). Blockage of NPP by furosemide resulted in the pronounced inhibition of parasite growth in culture suggesting the indispensability of NPP in iRBCs (Staines et al., 2004). One of the channel molecules of NPP has recently been extensively characterized as a plasmodial surface anion channel (PSAC), a widely accepted iRBC channel protein shown to enhance nutrient permeability of iRBCs (Alkhalil et al., 2009; Desai, 2012; Kirk, 2015). While the enhanced permeability of iRBCs has been experimented out for decades, the molecular identity of PSAC was largely unknown until recently, when a cytoadherence‐linked antigen 3 (Clag3) has been shown to be the key player of the channel for nutrient uptake (Alkhalil et al., 2009; Desai, 2012, 2014; Gupta et al., 2018; Nguitragool et al., 2011). In the formation of PSAC, Clag3 protein forms a homodimer, and RhopH2 and RhopH3 do associate with the dimer for the construction of functional channel (Counihan et al., 2017; Gupta et al., 2018; Ito et al., 2017; Kaneko et al., 2005; Nguitragool et al., 2011; Schureck et al., 2021; Sherling et al., 2017). Under PTEX suppressed condition, Clag3 still translocate into the host cell suggesting an alternative mechanism of Clag3 export but in the PTEX suppressed parasite lines, the transport of solutes by PSAC was diminished (Beck et al., 2014; Comeaux et al., 2011) indicating that other exported proteins are required for channel formation either in association with or independent of Clag3.

Based on experimental evidence, the non‐ribosomal role (s) of oligomeric/tetrameric P2 on the iRBC surface appears to be important but currently is at the stage of speculation. The tight regulation of oligomerization and subsequent localization of oligomer/tetramer on the iRBC surface at the trophozoite stage drives the attention towards its direct possible role in the formation of some channel either in association with Clag3 or independently for small molecule transport. P2 has one putative transmembrane (TM) domain from amino acid N’64‐84C’ but in the oligomeric state how this TM domain is important for host membrane insertion is currently elusive. Immunofluorescence assay (IFA) at ​the trophozoite stage showed Clag3 on iRBC surface co‐localized with RhopH3 (Sherling et al., 2017). But at this stage of trophozoite, P2 oligomers were diminished as IFA using E2G12 did not stain the iRBC surface suggesting that oligomeric P2 may not be a component of PSAC but there is a possibility of channel formation by P2 oligomers either in association with other export proteins or independently. If oligomeric P2 is forming a channel with/without other parasitic proteins, then this channel does not seem to complement the function of PSAC under the null state of Clag3.1 and Clag3.2 as these null parasites showed significant growth inhibition (Gupta et al., 2018; Kaneko et al., 2005; Nguitragool et al., 2011), hence there is a possibility that PSAC and putative channel of oligomeric P2 both are working independently.

Oligomeric/tetrameric P2 being molten globule in nature and having hydrophobic pockets on the surface also drives the speculation toward its possible direct interaction with hydrophobic molecules such as lipids. Parasite culture medium devoid of oleic acid and palmitic acid resulted in parasite cell cycle arrest (Mitamura et al., 2000) similar to P2 antibody‐mediated arrest (Das et al., 2012a). Hence, it could be logical to think and rationale to design experiments to validate the interaction of P2 oligomers with lipids which are already reported to be crucial for parasite progression. In addition to possible channel formation or P2‐lipid interaction, oligomeric P2 might have other non‐ribosomal function (s) depending on the interaction of P2 with other partner proteins on the iRBC surface and the structure of the entire oligomeric complex.

## 6 FRONTIER QUESTIONS

**FIGURE 1:**
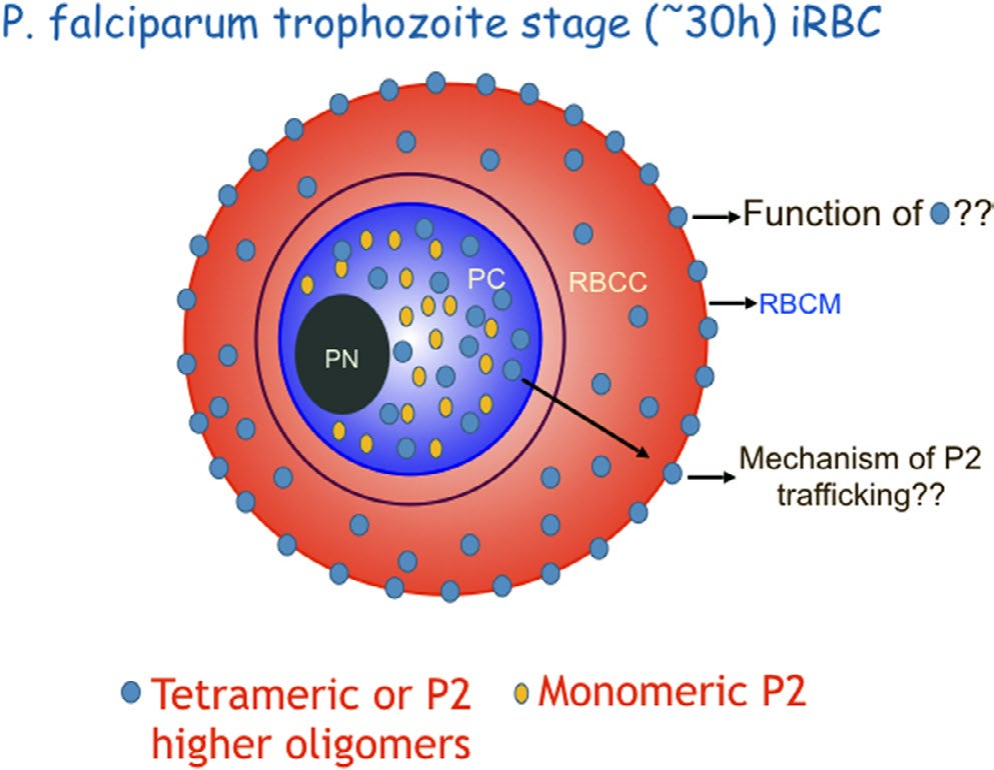
Cartoon of *Plasmodium* *falciparum*‐infected trophozoite stage iRBC depicting oligomeric states and localization of ribosomal P2 protein in different compartments of iRBC raising several critical questions portrayed in the diagram. PC, parasite cytoplasm; PN, parasite nucleus; RBCM, red blood cell membrane; RBCC, red blood cell cytoplasm

Finding oligomeric parasite P2 protein on the iRBC surface was puzzling but at the same time raises the possibility to target it. Unraveling the function of P2 on the iRBC surface could open several avenues towards our effort to develop novel anti‐malarial small molecules and might also come under consideration as a possible vaccine candidate as recently reported (Szuster‐Ciesielska et al., 2019). Understanding a fundamental non‐ribosomal role (s) of a ribosomal protein in the propagation of *Plasmodium* parasites in red blood cells can be harnessed to develop new strategies to target this highly resilient parasite and will unmask novel biochemical pathways operational at the trophozoite stage of parasite propagation. Based on current understanding, below there are three key unresolved questions depicted in Figure 1 that need to be answered to understand the eccentric P2 cell biology in *Plasmodium* parasites, and toward that, my laboratory is fully engaged.

(1) What is the function of oligomeric/tetrameric P2 on the iRBC surface at the late trophozoite/early schizogonic stage?

(2) What is the mechanism of P2 translocation from the parasite cytoplasm to the iRBC surface?

(3) What is the nature of P2 oligomers on the iRBC surface? Does it form a channel complex?

## 7 CONCLUSIONS

Cell division checkpoints and non‐PEXEL protein export in *Plasmodium falciparum*‐infected human RBCs are the two aspects that need to be pondered in apicomplexan cell biology. Here, we have summarized the uncanny nature of a parasite ribosomal stalk protein P2 which appears to translocate to the infected red blood cell (iRBC) surface and seems to be involved in the regulation of parasite nuclear division at the initial stage. P2 being a non‐PEXEL protein, requires prior oligomerization before trafficking to the iRBC surface which indicates that for non‐PEXEL protein export, oligomerization might be one of the prerequisite steps in the cascade of molecular events of non‐PEXEL protein trafficking in iRBCs.

Functional inhibition of P2 oligomers on the iRBC surface either by using monoclonal antibodies or by genetic knockdown resulted in the nuclear division arrest of the parasites which possibly suggests that the P2 oligomers might be involved as a checkpoint regulator or simply as a gatekeeper at the initial stage of nuclear division. Oligomeric P2 on the iRBC surface appears to form a channel protein complex either independently or in association with other parasite proteins to perform a regulatory role during parasite nuclear division. These serendipitous findings of the uncanny nature of P2 protein in malaria parasites offer to explore more about non‐ribosomal indefensible functions of ribosomal proteins in apicomplexan parasites and certainly, the molecular role of P2 oligomers in the regulation of parasite nuclear division are being looked at towards a better fundamental understanding about cell division checkpoints and the possibility to inhibit the function of P2 oligomers using small molecules in iRBCs.

## CONFLICT OF INTEREST

None declared.

​

ETHICS STATEMENT

None required.

## AUTHOR CONTRIBUTION

**Sudipta Das:** Conceptualization (lead); Formal analysis (lead); Funding acquisition (lead); Resources (lead); Supervision (lead); Writing‐original draft (lead); Writing‐review & editing (lead). **Bhaskar Roy:** Writing‐original draft (supporting); Writing‐review & editing (supporting). **Saswata Chakrabarty:** Writing‐original draft (supporting); Writing‐review & editing (supporting).

## Data Availability

Not applicable.
